# SARS-CoV-2 spike glycosylation affects function and neutralization sensitivity

**DOI:** 10.1101/2023.06.30.547241

**Published:** 2023-06-30

**Authors:** Fengwen Zhang, Fabian Schmidt, Frauke Muecksch, Zijun Wang, Anna Gazumyan, Michel C. Nussenzweig, Christian Gaebler, Marina Caskey, Theodora Hatziioannou, Paul D. Bieniasz

**Affiliations:** 1 Laboratory of Retrovirology, The Rockefeller University, New York, NY 10065, USA.; 2 Current address: King Abdullah University of Science and Technology, Thuwal, Makkah, Saudi Arabia. Center for Integrative Infectious Disease Research, Universitätsklinikum Heidelberg, 69120 Heidleberg, Germany.; 3 Laboratory of Molecular Immunology, The Rockefeller University, New York, NY 10065, USA.; 4 Current address: Laboratory of Translational Immunology of Viral Infections, Charité - Universitätsmedizin Berlin, Germany.; 5 Howard Hughes Medical Institute, The Rockefeller University, New York, NY 10065, USA.

## Abstract

The glycosylation of viral envelope proteins can play important roles in virus biology and immune evasion. The spike (S) glycoprotein of severe acute respiratory syndrome coronavirus-2 (SARS-CoV-2) includes 22 N-linked glycosylation sequons and 17 O-linked glycosites. Here, we investigated the effect of individual glycosylation sites on SARS-CoV-2 S function in pseudotyped virus infection assays and on sensitivity to monoclonal and polyclonal neutralizing antibodies. In most cases, removal of individual glycosylation sites decreased the infectiousness of the pseudotyped virus. For glycosylation mutants in the N-terminal domain (NTD) and the receptor binding domain (RBD), reduction in pseudotype infectivity was predicted by a commensurate reduction in the level of virion-incorporated spike protein. Notably, the presence of a glycan at position N343 within the RBD had diverse effects on neutralization by RBD-specific monoclonal antibodies (mAbs) cloned from convalescent individuals. The N343 glycan reduced overall sensitivity to polyclonal antibodies in plasma from COVID-19 convalescent individuals, suggesting a role for SARS-CoV-2 spike glycosylation in immune evasion. However, vaccination of convalescent individuals produced neutralizing activity that was resilient to the inhibitory effect of the N343 glycan.

## Introduction

Severe acute respiratory syndrome coronavirus 2 (SARS-CoV-2) is the causative agent of the COVID-19 disease and has caused a devastating pandemic (1, 2). SARS-CoV-2 encodes a spike (S) glycoprotein which binds angiotensin-converting enzyme 2 (ACE2) and mediates viral entry into host cells (3–6). The S glycoprotein (1273 aa) consists of a signal peptide followed by the S1 subunit (13–685 aa) and the S2 subunit (686–1273 aa). These two subunits are separated by a furin cleavage site (PRRAR), abrogation of which can increase virus infectivity in some circumstances (7). The receptor-binding domain (RBD, 319–541 aa) that is responsible for ACE2 binding (6) and the N-terminal domain (NTD), both encoded in S1, are the major targets of neutralizing antibodies. Like other viral envelope glycoproteins including HIV-1 (8), SARS-CoV-2 spike protein is heavily glycosylated (9–11). Indeed, approximately 40% of the surface the SARS-CoV-2 S protein expressed in human 293T cells is shielded by glycans (12). The majority of this shield is comprised of N-linked oligomannose-type or complex glycans, linked to 22 sites (Asn-X-Ser/Thr) on the S protein (13). Additionally,17 O-linked glyco-sites have been identified by biochemical methods (14–16).

Glycosylation of viral envelope proteins can play an important role in virus-host interactions (17). In the case of HIV-1, for example, N-linked glycans are essential for correct folding and processing of gp120 as well as structural rearrangements required for receptor binding (18). The HIV-1 glycan shield also plays a crucial role in preventing neutralizing antibodies from binding to HIV-1 envelope (19). Likewise, S glycosylation affects SARS-CoV-2 infection (20). Blocking N-glycan biosynthesis onto SARS-CoV-2 spike protein and, to lesser extent, O-glycan elaboration, reduces viral infectivity (21). Additionally, cryo-electron microscopy studies have revealed that the N-glycan at position N343 in the RBD facilitates transition of the spike protein to the ‘open’ conformation, which is important for ACE2 binding (22). Accordingly, mutation of this site (N343Q) reduced viral entry into ACE2-expressing cells (23).

Little is known about how SARS-CoV-2 S glycosylation might affect immune surveillance. It is conceivable that glycans sterically shield underlying epitopes from recognition by antibodies, as is the case in HIV-1 (19). Many SARS-CoV-2 neutralizing antibodies target the RBD and can be divided into 4 broad classes based on the epitopes targeted (24). Class 1 antibodies recognize epitopes overlapping with the ACE2-binding site and bind only to ‘up’ RBDs. Class 2 antibodies bind both ‘up’ and ‘down’ RBDs and also block ACE2 binding. In contrast, class 3 antibodies bind epitopes distinct from the ACE2 binding site but can potently neutralize. Class 4 antibodies are generally less potent and recognize epitopes that are distinct from the ACE2 binding site that are shielded in the down conformation. Interestingly, some antibodies, namely S309 and SW186, recognize epitopes that include the N343 glycan (25) (26), raising the possibility that this glycan might play a dual role in antibody recognition, either as shield or as a component of an epitope.

Co-expression of SARS-CoV-2 S with envelope-deficient viruses such as HIV-1 (human immunodeficiency virus-1) produces pseudotyped viruses capable of infecting ACE2-expressing cells and is widely used as a surrogate to study viral entry and neutralization by antibodies (27, 28). To comprehensively understand the role of spike glycans in viral infectivity and antigenicity, we individually mutated each of the 22 N-linked glycosylation sites in the spike protein as well as two O-linked sites (S323 and T325) in the RBD. We found mutations introduced at many glycosylation sites in the NTD and RBD reduced pseudotype infectivity, and the magnitude of this effect was predicted by the magnitude of the loss of S incorporation into virions. Furthermore, while the S protein levels in virions had little effect on neutralization sensitivity, the presence or absence of a glycan on N343 in RBD governed the sensitivity to some monoclonal antibodies cloned from convalescent individuals. Moreover, the glycan at N343 reduced neutralization sensitivity to polyclonal antibodies from convalescent individuals, but this evasive effect imparted by glycosylation was overcome by plasma antibodies from the same individuals who were subsequently vaccinated.

## Results

### Levels of virion-incorporated SARS-CoV-2 spike protein and pseudotyped HIV-1 particle infectiousness

**FIG 1:**
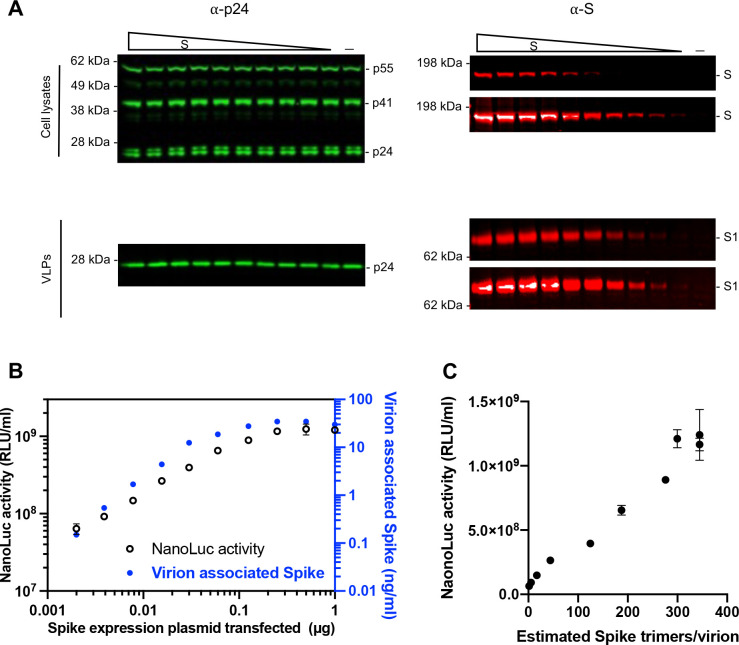
The effect of spike proteins level on pseudotype infectiousness (A) Western blot analysis of 293T cell lysates (upper panel) or virions (lower panel) at 48 hours after transfection with various amounts (0 ng, 2 ng, 3.9 ng, 7.8 ng, 15.6 ng, 31.2 ng, 62.5 ng, 125 ng, 250 ng, 500 ng, or 1000 ng) of a WT SARS-CoV-2 spike expression plasmid along with envelope-deficient HIV-1 proviral plasmid expressing NanoLuc luciferase. Each S blot was scanned twice, at low intensity (*upper*) and high intensity (*low*er), respectively. Representative of three independent experiments. (B) Characterization of virions pseudotyped with spike expression plasmids. Infectiousness (on left y axis) was quantified by measuring NanoLuc luciferase activity (RLUs) following infection of 293T cells expressing ACE2 (293T/ACE2.cl22) in 96-well plates with pseudotyped viruses. The S1 incorporated into viruses (on right y axis) was determined by quantitative Western Blot, using purified recombinant S-6P-NanoLuc proteins as standard, representative of three independent experiments. The mean and range of two technical replicates are shown. (C) Characterization of virions pseudotyped with spike expression plasmids as in (B). virus infectiousness (on y axis) was plotted against S trimers per virion (on x axis), which was determined by quantitative Western blot, using purified recombinant S-6P-NanoLuc proteins as standard. Representative of three independent experiments.

While SARS-CoV-2 S pseudotyped HIV-1 has been widely utilized to study S-mediated viral entry and neutralizing activities by antibodies, the extent to which the amount of S protein on virions affects particle infectivity and neutralization sensitivity is not fully understood. To address this question, we co-transfected 293T cells with various amounts of an S expression plasmid (pSARS-CoV-2_Δ19_), along with an envelope-deficient HIV-1 proviral plasmid encoding NanoLuc. The number of SARS-CoV-2 spikes incorporated into HIV-1 pseudotype virions were estimated using purified recombinant purified S protein (S-6P-NanoLuc (29)) and recombinant p24 CA proteins as standards on near-IR fluorescent western blotting (LiCor) and assuming 1500 capsid (CA) protein subunits per mature HIV-1 particle (30, 31) (Fig. S1A). While the amount of S expression plasmid used for transfection had little effect on the amount of virions produced (Fig. 1A), the amount of S protein incorporated into virions correlated with the amount expressed in cells. S incorporation into virions reached a plateau (~30 ng S/ml in virions, or around 300 spike trimers per virion) when 0.125 μg or 0.25 μg of an S expression plasmid was co-transfected with the HIV-1 proviral plasmid (Fig. 1A, 1B, 1C).

To assess the effect of the number of spikes on pseudotype infectiousness, the titers of pseudotyped viruses were measured on 293T cells expressing ACE2 (293T/cl.22). Co-transfection with as little as 2 ng of S expression plasmid produced virions that induced 1000x the level of luciferase observed with ‘bald virions’, indicating that small amounts of S protein (0.15 ng/ml or a mean of ~1–2 S trimers per virion) were sufficient to mediate viral entry (Fig. S2, Fig. 1B and 1C). Nevertheless, pseudotype virion infectivity increased with increasing spike numbers. Indeed, infectivity and the number of spikes was approximately linearly correlated, in the range 1 spike to 300 spikes per virion (Fig. 1C). Of note, these spike numbers are comparable to the average number of S trimers on authentic SARS-CoV-2 virions (25–127 prefusion spikes per virion) (32) and higher than the numbers of gp120 trimers on HIV-1 virions (33).

### Reduced infectious virion yield conferred by SARS-CoV-2 spike glycosylation site removal

**FIG 2:**
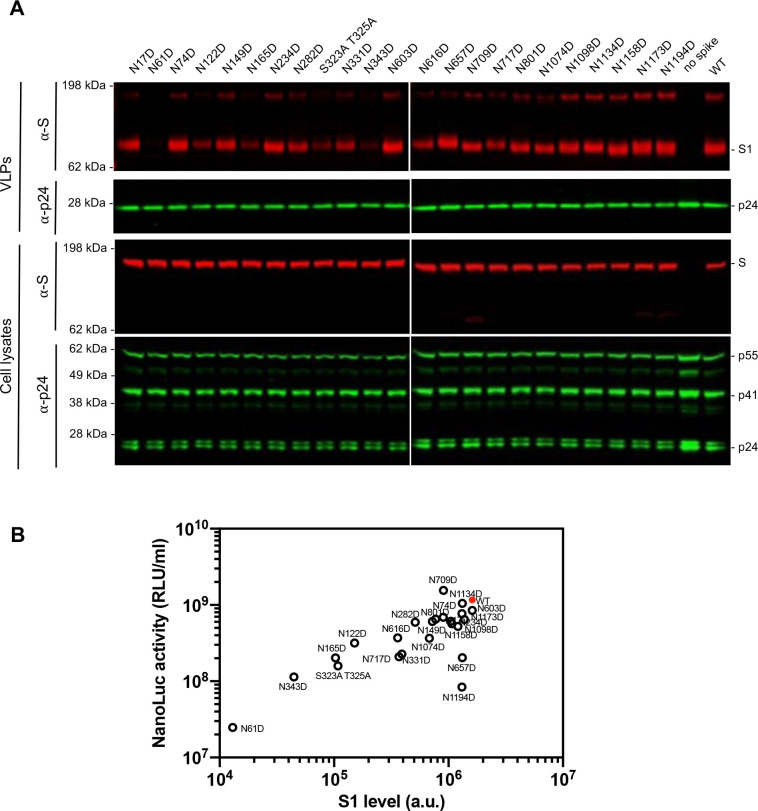
The impact of glycosylation site mutations on spike incorporation and particle infectivity (A) Western blot analysis of 293T cell lysates (lower panel) or virions (upper panel) at 48 hours after transfection with 1 μg of glycosylation site mutants or wild-type spike expression plasmid along with envelope-deficient HIV-1 proviral plasmid expressing NanoLuc. Representative of two independent experiments. (B) Characterization of virions pseudotyped with spike expression plasmids. Infectiousness (on y axis) was quantified by measuring NanoLuc luciferase activity (RLUs) following infection of 293T/ACE2.cl22 cells in 96-well plates with pseudotyped viruses. The S1 incorporated into viruses (on x axis) was determined by quantitative Western blot. Glycosylation site mutants are shown in black and wild-type spike is shown in red. The mean and range of two technical replicates are plotted.

To investigate the contribution of glycosylation to S protein function, we generated 22 spike substitution mutants, each containing a single Asn to Asp (N to D) substitution at one of the 22 N-glycosylation sequons. Additionally, we generated a mutant with Ala replacements at potential O-linked sites S323 and T325 in the receptor binding domain (RBD) (13). None of these substitutions affected the levels of the S protein in transfected cells, but some of them reduced S incorporation into virions (Fig. 2A). Specifically, N61D, N122D, N165D, N343D, and S323A T325A that fall within S1, that includes the NTD and RBD, exhibited 10-fold or greater reductions in the levels of virion-associated S protein, suggesting that these glycans affect S protein transport or virion incorporation. Conversely, removal of the glycosylation sites in S2 had either no or minor effects on S protein incorporation into virions (Fig. 2A). Measurements of the yield of infectious pseudotyped particles carrying each of the substitutions indicated that several substitutions in the NTD and RBD markedly reduced infectivity. For example, the N61D substitution reduced infectivity by almost 50-fold (Fig. 2B, Fig. S2) while substitutions at glycosylation sites in the RBD, namely N331D, N343D, and S323A T325A, resulted in 5- to 10-fold reduction in infectivity (Fig. 2B, Fig. S2). Western blot analyses revealed that the amount of S protein in virions was directly correlated with infectivity (Fig. 2B), suggesting a potential role for most glycans during synthesis or folding of spike trimers, or their incorporation into virions, rather than in S protein function after virion incorpration. In contrast, two substitutions (N1194D and N657D) did not change the amount of S protein in virions, but substantially reduced infectivity (Fig. 2B, Fig. S2). This finding suggests a functional role for N1194 and N657 glycans in spike conformation or stability on virions, or function during viral entry. Overall, we conclude that a subset of glycosylation sites is important for the incorporation of S protein into virions, while others are required for optimal particle infectivity.

### Impact of spike density and RBD glycosylation on neutralization sensitivity

**FIG 3:**
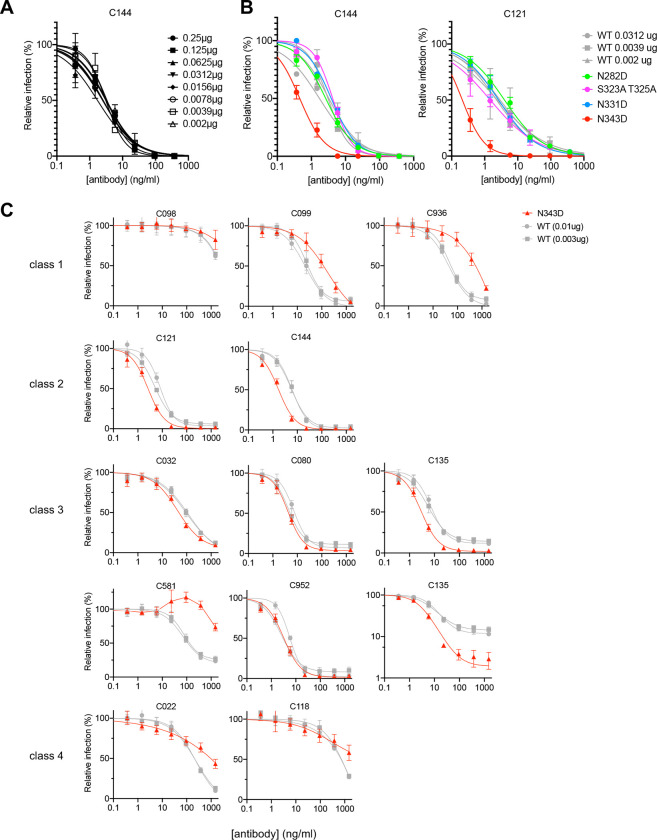
Neutralization sensitivity of Spike to monoclonal antibodies is affected by N343D substitution (A) Quantification of pseudotyped virions from cells transfected with various amounts of wild-type spike expression plasmid along with envelope-deficient HIV proviral plasmid expressing NanoLuc in the presence of the indicated concentrations of the class 2 neutralizing monoclonal antibody C144. Infectivity was quantified by measuring NanoLuc luciferase levels (RLU). The mean and range of two technical replicates are shown. (B) Quantification of glycosylation site mutant N282D, S323A T325A, N331D, and N343D or wild-type spike pseudotyped virus infection in the presence of the indicated concentrations of the class 2 neutralizing monoclonal antibody C144 (*left*) or C121 (*right*). Infectivity was quantified by measuring NanoLuc luciferase levels (RLU). The mean and range of two technical replicates are shown. (C) Quantification of neutralization of glycosylation site mutant N343D in the background of furin uncleavable (R683G) SARS-CoV-2 S pseudotyped virus infection in the presence of the indicated concentrations of a panel of monoclonal antibodies, including class 1 (C098, C099, and C936), class 2 (C121 and C144), class 3 (C032, C080, C135, C581, and C952), and class 4 (C022 and C118) antibodies. As controls, glycosylation intact spike expression plasmid (WT in the R683G background) was transfected at two doses, 10 ng or 3 ng, and the neutralization sensitivity of the resulting viruses were assessed in parallel. The mean and range of two technical replicates are shown. The C135 neutralization graph is depieced with both a linear and logarithmic Y-axis to more clearly show effects of glycosylation on the completeness of neutralization at high antibody concentrations

he RBD is the major target of neutralizing antibodies. To determine the effect of glycan removal on sensitivity to neutralizing antibodies, we focused on glycosylation sites in the RBD (N331 and N343), and one site adjacent to the RBD (N282). Because these glycosylation sites affected the level of spike incorporation into virions, we first tested the effect of SARS-CoV-2 spike density on neutralization sensitivity, as this parameter could be a potential confounder. We harvested pseudotyped virions from cells transfected with varying amounts (from 2 ng to 1 μg) of wild-type S expression plasmid and tested their sensitivity to C144, a potent class 2 neutralizing human monoclonal antibody cloned from a convalescent individual (34) (Fig. 3A). Notably, varying the levels of WT S protein on virions over a wide range (Fig. 1B) had no discernable effect on neutralization sensitivity to C144.

We next tested the neutralization sensitivity of the RBD glycosylation site mutants bearing Asn to Asp mutation at N-linked sites (N331D and N343D) or proximal (N282D) to the RBD or alanine substitutions at O-linked sites S323 and T325. To provide matched control viruses with approximately similar numbers of S trimers and similar levels of infectivity, neutralization of the glycosylation site mutants was compared to virions generated with an appropriate level of the WT spike protein. We compared the neutralization sensitivity of WT and mutant virions to C144 and another potent class 2 neutralizing antibody, C121, both of which target the ACE2 binding site. While the N282D, S323/T325A, and N331D mutant pseudotypes exhibited neutralization sensitivities that were similar to the WT pseudotype, the N343D substitution conferred significantly increased neutralization sensitivity to both C144 and C121 (Fig. 3B). Specifically, the half maximal inhibitory concentration (IC_50_) of C144 was reduced from 1.8 to 3.4 ng/ml (against the WT pseudotype) to 0.45 ng/ml (against the N343D pseudotype), while the C121 IC_50_ was reduced from 2.3 to 2.9 ng/ml (against the WT pseudotype) to 0.21 ng/ml (against the N343D pseudotype).

### Positive and negative effects of RBD glycosylation on sensitivity to human monoclonal antibodies

To determine the effects of glycosylation more broadly on SARS-CoV-2 sensitivity to neutralizing antibodies, we used pseudotyped viruses bearing spike proteins with R683G substitution, which ablates the furin cleavage site. This substitution does not affect S incorporation into virions (Fig. S1B) but enhances particle infectivity (7). The glycosylation site mutations had a smaller effect on spike incorporation into virions in the R683G context (Fig. S3A). Nevertheless, transfection of cells with 1 μg of N282D, S323A T325A, and N331D S expression plasmids generated pseudotyped viruses with S protein amounts and infectious properties comparable to those generated by transfection of 30–100 ng of WT S expression plasmid (Fig. S3A and S3B). Transfection of cells with 1 μg of the N343D spike expression plasmid yielded pseudotyped virus particles carrying a similar amount of S protein to those generated by cells transfected with 10 ng of the R683G S protein expression plasmid (Fig. S3A), while the particle infectivity was similar to those from cells transfected with 3 ng of the WT R683G expression plasmid (Fig. S3B). To evaluate the effect of glycosylation site mutations on sensitivity to neutralizing antibodies, we generated WT and mutant virion stocks bearing similar amounts of spike protein and then assessed their susceptibility to neutralization by a panel of RBD-specific human monoclonal antibodies of the various neutralizing classes (24) recovered from convalescent individuals, including antibodies from class 1 (C098 (35), C099 (35), C936 (36)), class 2 (C121 (34), C144 (34)), class 3 (C032 (34), C080 (35), C135 (34), C581 (36), C952 (24)), and class 4 (C022 (34) (37), C118 (34) (37)).

Of class 1 antibodies, C098 had only weak neutralizing activity, whereas its clonal relative C099 was potent (IC_50_ =21.3 ng/ml against WT (10ng) or 24.5 ng/ml against WT (3ng) and its activity was resilient to many naturally occurring mutations in the RBD (7). The N343D mutation decreased the neutralization sensitivity to C099 by 7-fold, ie IC_50_ was increased to 176.9 ng/ml (Fig. 3C). Neutralization sensitivity to a third unrelated class 1 antibody, C936, was reduced by almost 10-fold, and IC_50_ was increased from 41.1 ng/ml against WT (10ng) or 54.9 ng/ml against WT (3ng) to 467.1 ng/ml against N343D (Fig. 3C).

In contrast to the class 1 antibodies, pseudotyped virus sensitivity to two class 2 antibodies was increased for the N343D mutant compared to WT. As was the case in the context of the furin-cleavable S protein (Fig. 3B), the N343D mutation in R683G spike increased neutralization sensitivity to C121 and C144. The IC_50_ of C121 was reduced from 4.8 to 8.1 ng/ml (against the WT) to 2.6 ng/ml (against the N343D), while the C144 IC_50_ was reduced from 5.7 to 6.1 ng/ml (against the WT) to 1.7 ng/ml (against the N343D) (Fig. 3C).

Class 3 antibodies, which bind epitopes distinct from the ACE2 binding site, showed a complicated pattern of effects in response to the N343D substitution. Some class 3 antibodies, including C032, C080, and C952, inhibited the WT and N343D mutant pseudotypes with approximately the same potency. A different class 3 antibody C135, that exhibited incomplete neutralization of the WT pseudotypes at high concentrations despite exhibiting low IC_50_ (IC_50_= 8.3–11.5ng/ml), was able to achieve almost complete neutralization of the N343D pseudotypes at high concentrations (Fig. 3C). In contrast, another class 3 antibody C581 showed reduced potency against the N343D mutant, ie, the IC_50_ was >2000 ng/ml against N343D compared to 70.0 to 76.6 ng/ml against the wild-type pseudotype (Fig. 3C).

For two class 4 antibodies, C022 and C118, the N343D substitution affected the slope of the neutralization curves and conferred partial resistance at high of antibody concentrations (Fig. 3C). For example, C022 at 2000 ng/ml almost completely neutralized the wild-type pseudotypes but only inhibited the N343D pseudotypes by ~50%. A similar trend was seen for a second class 4 antibody, C118.

**FIG 4:**
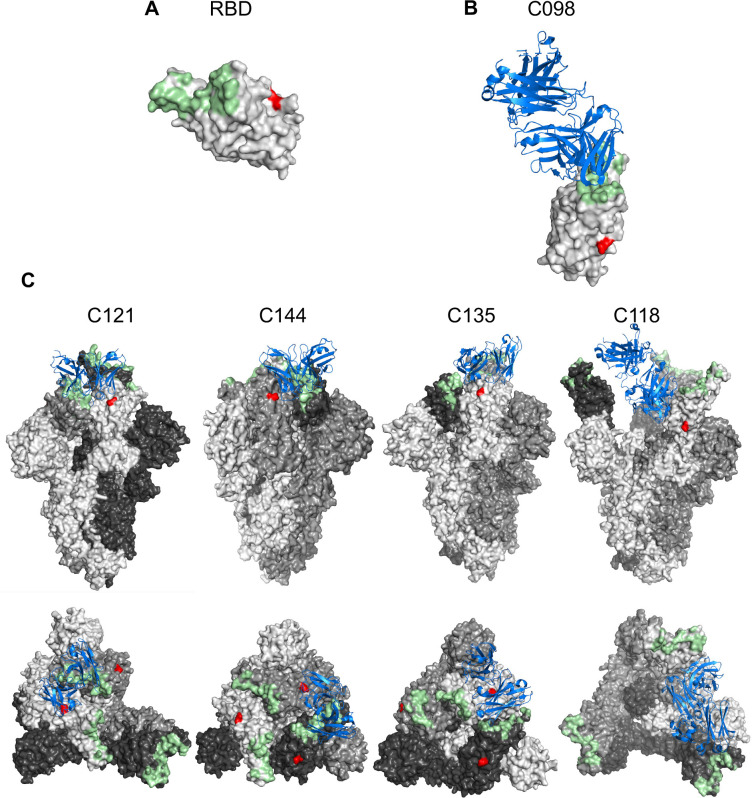
Proximity of N343 to antibody binding sites (A) Surface representation of the receptor-binding domain (RBD) in an X-ray crystal structure (PDB ID: 7K8M). ACE2-binding site and the N343 glycosylation site are highlighted in palegreen and red, respectively. (B) Views of antibody Fab variable domains (blue) binding to spike (grey), in which each trimer subunit, is shown with distinct gray shade. ACE2-binding site and N343 are highlighted in pale green and red, respectively. The structures illustrated herein are C098 (PDB ID: 7N3I), C121 (PDB ID: 7K8X), C144 (PDB ID: 7K90), C135 (PDB ID: 7K8Z) and C118 (PDB ID: 7RKV).

Overall, the N343D substitution had a range of positive and negative effects on neutralization sensitivity that varied greatly dependent on the nature of the particular monoclonal antibody tested. To better understand the molecular basis for the impact of N343 glycan on antibody neutralization, we inspected the structures of some of the aforementioned antibodies in complex with spike (Fig. 4A, B, C). The glycosylation site at N343 (shown in red in Fig. 4A, B, C) is distinct from the binding site of the class 1 antibody C098 – suggesting that the effects of the glycan on sensitivity to class 1 antibodies are mediated through effects of the glycan on RBD conformational dynamics and epitope exposure (Fig. 4B). In contrast, for the class 2 antibodies C121 and C144, N343 is proximal to the antibody bound to the neighboring spike subunit (Fig. 4C). Since removal of the glycan increased sensitivity to these antibodies (Fig. 3C), it is likely that the N343 glycan partly shields these class 2 antibody epitopes. For class 3 antibodies, N343 protrudes towards the C135 binding site on the same S subunit (Fig. 4), potentially explaining incomplete neutralization by this antibody (Fig. 3C). For the class 4 antibody C118, N343 is distal to the antibody binding site on spike (Fig. 4C), suggesting that N343 glycan is not involved in direct antibody binding and likely exerts changes in neutralization through effects on RBD conformational dynamics.

Other RBD substitutions (S323A/T325A and N331D) did not alter the neutralization sensitivity to most monoclonal antibodies tested herein (Fig. S4, Fig. S5). However, the N282D substitution, which lies outside the RBD, had marginal effect on sensitivity to the class I antibodies C099, C936 and the class 3 antibody C581 (Fig. S6).

### Effect of the N343 glycan on neutralization by polyclonal SARS-CoV-2 neutralizing plasma

**FIG 5:**
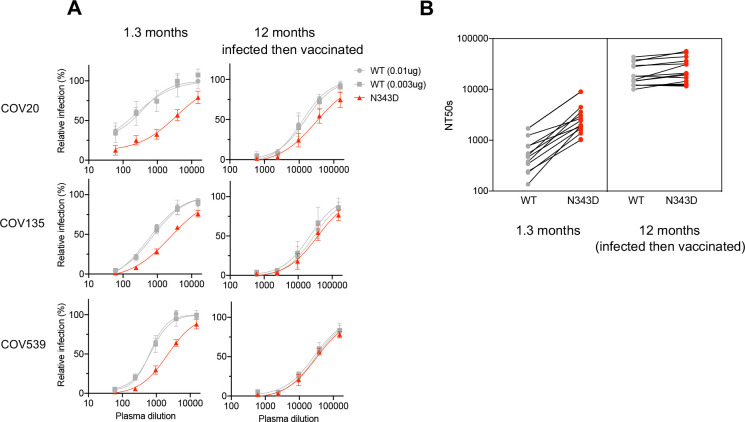
Neutralization sensitivity of N343D mutant to convalescent plasma (A) Plasma neutralization of N343D or glycosylation site intact spike (in the furin uncleavable (R683G) background) pseudotyped virus using 293T/ACE2.cl22 target cells. The convalescent plasma samples were collected at 1.3 months and 12 months (infected then vaccinated) and diluted four-fold serially followed by incubation with viruses. As controls, glycosylation intact spike expression plasmid (WT in the R683G background) was transfected at two doses, 10 ng or 3 ng, and the neutralization sensitivity of the resulting viruses were assessed in parallel. The mean and range of two technical replicates are shown. (B) Comparison of NT_50_ values for each of the 15 convalescent plasma samples collected at 1.3 months and at 12 months (infected then vaccinated) for the N343D or glycosylation intact spike pseudotypes.

Given that the N343D substitution exhibited different effects on sensitivity to neutralizing monoclonal antibodies, we next asked whether this substitution affected neutralization by polyclonal antibodies present in convalescent plasma. As with the monoclonal antibodies, we compared the neutralization sensitivity of N343D pseudotyped particles with that of WT pseudotyped particles containing same amount of WT spike protein or showing the same infectivity as the N343D pseudotype, to convalescent plasma from 15 patients collected early in the COVID19 pandemic (at 1.3 months after infection) and from the same individuals ~1 year later following subsequent vaccination (34) (36). The N343D mutant pseudotypes were more sensitive than the WT pseudotypes to convalescent plasma collected at 1.3 months after infection (Fig. 5A and Fig. S7). Indeed, the 50% neutralization titers (NT50) were a mean of 5.1-fold greater for the N343D mutant as compared to WT pseudotypes (p=0.0022) (Fig. 5B). Notably, the difference in neutralization sensitivity between N343D mutant (mean NT50=26179) and WT S pseudotypes (mean NT50=20853) was negligible (p=0.2805) when plasmas collected from the same individuals 1 year later and after subsequent vaccination were tested. Of note, these subsequently collected plasma exhibited higher neutralization potency than those collected shortly after infection (Fig. 5). We conclude that the glycan at N343 confers protection against neutralization by antibodies generated shortly after SARS-CoV-2 infection but that this effect is lost against antibodies from the same individuals who are subsequently vaccinated.

## Discussion

While SARS-CoV-2 spike pseudotyped viruses have been widely used as a surrogate to study S-mediated viral entry (28) little is known about the varying effect of S level on virions on particle infectivity and neutralization sensitivity. Previously it was reported that approximately 8 HIV-1 trimer-receptor interactions are required for HIV-1 to infect a target cell (38), a result that is broadly consistent with our finding that a small number (minimally an average of 1–2 spike per virion) is sufficient for detection of SARS-CoV-2 pseudotype infection. To achieve neutralization, monoclonal antibodies must encounter prefusion spikes and reduce the number of functional spike trimers below the threshold required for infection. We found that an increased level of S on pseudotyped virions is associated with increased infectivity but had little effect on neutralization sensitivity to monoclonal antibodies. This result suggests that monoclonal antibodies are in excess over functional spikes in a typical neutralization assay, and their measured potency is limited by their affinity, rather than by the amount of functional spike protein in a pseudotype neutralization assay.

In this study, we found that glycosylation at several sites in the spike NTD and RBD, including N61, N122, N165, N331, N343 and S323 T325 are required for full pseudotyped virus infectivity. Substitution of these glycosylation sites resulted in a reduction in pseudotype infectivity of about 10-fold or more and correlated with a reduction in spike incorporation into virions. While it is unclear precisely how glycosylation on these sites drives S incorporation into virions, glycans could in principle affect protein stability and trafficking through the secretory pathway (39). We note, however, that the glycosylation site mutations did not affect steady state levels of S in transfected cells. Compared with other glycosylation sites in the S1 domain, these sites are highly conserved among sarbecovirus S proteins (40). Likewise, the conservation of these glycosylation sites is observed among the major SARS-CoV-2 variant lineages, including Alpha, Beta, Gamma, Delta and Omicron (41). Some studies, largely employing molecular dynamics simulations, have suggested glycans on spike proteins affect the conformational dynamics of the spike’s RBD “up” and “down” states (10) (42). In particular, the glycan at N343 stabilizes RBD states in a process termed “glycan gating” (22, 43, 44). Our findings show that the N-glycan at N343 affected incorporation into virions, suggesting that the conformational state, might affect trimer assembly or stability, transit through the secretory pathway or incorporation into pseudotyped virions.

The N343 glycan also affected neutralization by RBD-targeted monoclonal antibodies, and the effect was largely dependent on the class to which antibodies belong. The N343 glycan is positioned on the RBD distal to the ACE2 and class 1 antibody (C098) binding site, but increased sensitivity to class 1 antibodies (C098, C099, C936), which bind only to “up” RBDs (24), suggesting that this effect is mediated through the impact of the glycan on RBD conformation. Conversely, the N343 glycan reduced sensitivity to class 2 antibodies, including C121 and C144, both of which can bind both ‘up’ and ‘down’ RBDs. Cryo-EM structures (24) (Fig. 4) show that N343 is located proximally to the C121 and C144 antibody bound to the neighboring subunit, in a manner that might interfere with antibody binding. Overall, our results are consistent with a model in which N343 glycan affects sensitivity to class 1 and class 2 antibodies by affecting the RBD conformational dynamics (43) and also potentially by sterically hindering class 2 antibody binding into neighboring RBDs in the “down” conformation (Fig. 4).

The N343 glycan is proximal to the binding sites of class 3 antibodies, and the N343D substitution had a range of effects on sensitivity to class 3 antibodies. Three clonally related antibodies, C032, C080 and C952, were unaffected by the N343D substitution, while substantial but opposing effects were seen for two others, C135 and C581. In the case of C135, removal of the glycan reduced the fraction of virions that resisted neutralization at high antibody concentration. A possible explanation for this phenomenon is that the N343 site is heterogeneously glycosylated, and some subfraction of the glycans occlude the C135 binding site. For C581, removal of the N343 glycan had the opposite effect, reducing neutralization sensitivity. In this case it seems likely C581 mimics the properties of two previously described cross-reactive class 3 antibodies, S309 and SW186, for which the N343 glycan constitutes part of the antibody binding site (25) (26). Finally, for the two class 4 antibodies, the glycan at N343 affected the character of the neutralization curve. Since in these cases the antibody binding site is on the opposite face of the RBD to that of the glycan, it is likely that these effects are mediated through alteration of spike conformational dynamics and exposure of the class 4 epitope, which is shielded in the RBD ‘down’ conformation.

The N343 glycan reduced neutralization by convalescent plasma collected at 1.3 months after infection, echoing the effects on sensitivity to the C121, C144 (class 2) and C135 (class 3) antibodies. Conversely, neutralization by convalescent plasma from the same individuals (collected at 12 months following subsequent vaccination) was unaffected by the N343 glycan. That neutralizing antibodies are relatively sensitive to glycan-mediated protection early after infection may be a reflection of the fact that the initial neutralizing response is based to a large extent on class 2 antibodies that are easily escaped (29, 34). Subsequent increases in antibody affinity and neutralizing potency and a shift in the RBD epitopes that are recognized, following months of affinity maturation and boosting by vaccination (7, 35, 36, 45) results in neutralizing antibodies that are mostly unaffected by the N343 glycan. In sum, while the SARS-CoV-2 N343 glycan affects both spike conformation and neutralization sensitivity shortly after infection, antibody evolution confers sufficient potency and breadth to combat glycan-mediated immune evasion.

## Materials and Methods

### Antibodies and recombinant HIV p24

Antibodies used here are anti-SARS CoV-2 spike antibody [1A9] (Genetax, GTX632604) and anti-HIV capsid p24 (183-H12-5C, NIH AIDS Research and Reference Reagent Program). Secondary antibodies included goat anti-mouse conjugated to IRDye 800CW or IRDye 680 for Western blot analysis. The recombinant HIV p24 protein was purchased from abcam (ab43037).

### Plasmid construction

The plasmid expressing C-terminally truncated, human-codon-optimized SARS-CoV-2 spike protein (pSARS-CoV-2_Δ19_) has been previously described (28). Using this plasmid as the template, asparagine at N-linked glycosylation sites was replaced by aspartate by overlap-extension PCR amplification with primers that incorporated the corresponding nucleotide substitutions. O-linked glycosylation sites in the RBD region (S323, T325) were replaced by alanine using the same strategy. The purified PCR products were then inserted into the pCR3.1 expression vector with NEBuilder^®^ HiFi DNA Assembly. Some mutations within or adjacent to the RBD region, including N282D, N331D, N343D, and S323A T325A, were also introduced in spike bearing the R683G substation which impairs the furin cleavage site and enhances particle infectivity.

### Cell lines

Human embryonic kidney HEK-293T cells (ATCC CRL-3216) and the derivative expressing ACE2, ie 293T/ACE2.cl22, were maintained in Dulbecco’s Modified Eagle Medium (DMEM) supplemented with 10% fetal bovine serum (Sigma F8067) and gentamycin (Gibco). All cell lines used in this study were monitored periodically to ensure the absence of retroviral contamination and mycoplasma.

### Generation of clarified SARS-CoV-2 pseudotyped virions and infectivity measurement

To generate HIV-1/NanoLuc-SARS-CoV-2 pseudotyped virions, two million 293T cells in 10-cm dish were transfected with 7.5 μg of HIV-1 proviral plasmids expressing NanoLuc along with increasing amounts (0 ng, 2 ng, 3.9 ng, 7.8 ng, 15.6 ng, 31.2 ng, 62.5 ng, 125 ng, 250 ng, 500 ng, or 1000 ng) of WT or 1000 ng of glycosylation site mutant SARS-CoV-2 expression plasmids (pSARS-CoV-2_Δ19_). In transfection experiment to generate viruses bearing R683G substitution, 1 ng, 3 ng, 10 ng, 30 ng, 100 ng, and 300 ng of expression plasmid were used instead. The total amount of DNA was held constant by supplementing the transfection with empty expression vector. Cells were harvested at 48 hr post transfection and subjected to Western blot analysis. Virus-containing supernatant was filtered (0.2 μm), and, to remove cell debris, clarified by Lenti-X (TaKaRa). Particle infectivity was measured as previously described (28). Briefly, viral stocks were three-fold serially diluted and added to 293T/ACE2 cl.22 in 96-well plates. Cells were then harvested at 48 hr post infection for measuring NanoLuc activity (Promega). The number of spike trimers per virion was estimated using the following formula: S ng/ml/78.3 × 1500/(p24 ng/ml/24)/3.

### Western blot analysis

Cell lysates were separated on NuPage Novex 4–12% Bis-Tris Mini Gels (Invitrogen). Proteins were blotted onto nitrocellulose membranes. Thereafter, the blots were probed with primary antibodies and followed by secondary antibodies conjugated to IRDye 800CW or IRDye 680. Fluorescent signals were detected and quantitated using an Odyssey scanner (LI-COR Biosciences).

### S-6P-NanoLuc protein purification

To express S-6P-NanoLuc proteins, Expi293 cells were transfected with S-6P-NanoLuc expression plasmids using ExpiFectamine 293 (ThermoFisher Scientific). Four days later, the supernatant was harvested and loaded on Ni-NTA agarose and, after thorough wash, S-6P-NanoLuc proteins were released after HRV 3C protease treatment.

### Neutralization assays

To measure neutralizing activity of monoclonal antibodies, serial dilutions of antibodies beginning at 3 μg/ml were four-fold serially diluted in 96-well plates over seven dilutions. To determine the neutralizing activity in convalescent plasma, the initial dilution started at a 1:30 (for plasma at 1.3 months) or a 1:150 (for plasma at 12 months). Thereafter, SARS-CoV-2 spike pseudotyped viruses were incubated with monoclonal antibodies or the convalescent plasma for 1 hr at 37°C in 96-well plates. The mixture was then added to 293T/ACE2cl.22 target cells seeded one day prior to infection so the final starting dilutions were 1.5 μg/ml for monoclonal antibodies and 1:60 or 1:300 for plasma. Cells were then harvested 48 hours post infection for NanoLuc luciferase assays.

### Human plasma samples and monoclonal antibodies

Monoclonal antibodies C022, C032, C080, C098, C099, C118, C121, C135, C144, C581, C936, and C952 used in this study were previously reported (24, 34–37). The human convalescent plasma samples (COVs) were obtained under protocols approved by Institutional Review Boards at Rockefeller University.

## Supplementary Material

Supplement 1

## References

[1] Zhu N, Zhang D, Wang W, Li X, Yang B, Song J, Zhao X, Huang B, Shi W, Lu R, Niu P, Zhan F, Ma X, Wang D, Xu W, Wu G, Gao GF, Tan W, China Novel Coronavirus I, Research T. 2020. A Novel Coronavirus from Patients with Pneumonia in China, 2019. N Engl J Med 382:727–733. 31978945 10.1056/NEJMoa2001017 PMC7092803

[2] Zhou P, Yang XL, Wang XG, Hu B, Zhang L, Zhang W, Si HR, Zhu Y, Li B, Huang CL, Chen HD, Chen J, Luo Y, Guo H, Jiang RD, Liu MQ, Chen Y, Shen XR, Wang X, Zheng XS, Zhao K, Chen QJ, Deng F, Liu LL, Yan B, Zhan FX, Wang YY, Xiao GF, Shi ZL. 2020. A pneumonia outbreak associated with a new coronavirus of probable bat origin. Nature 579:270–273. 32015507 10.1038/s41586-020-2012-7 PMC7095418

[3] Walls AC, Park YJ, Tortorici MA, Wall A, McGuire AT, Veesler D. 2020. Structure, Function, and Antigenicity of the SARS-CoV-2 Spike Glycoprotein. Cell 181:281–292 e6. 32155444 10.1016/j.cell.2020.02.058 PMC7102599

[4] Letko M, Marzi A, Munster V. 2020. Functional assessment of cell entry and receptor usage for SARS-CoV-2 and other lineage B betacoronaviruses. Nat Microbiol 5:562–569. 32094589 10.1038/s41564-020-0688-y PMC7095430

[5] Hoffmann M, Kleine-Weber H, Schroeder S, Kruger N, Herrler T, Erichsen S, Schiergens TS, Herrler G, Wu NH, Nitsche A, Muller MA, Drosten C, Pohlmann S. 2020. SARS-CoV-2 Cell Entry Depends on ACE2 and TMPRSS2 and Is Blocked by a Clinically Proven Protease Inhibitor. Cell 181:271–280 e8. 32142651 10.1016/j.cell.2020.02.052 PMC7102627

[6] Lan J, Ge J, Yu J, Shan S, Zhou H, Fan S, Zhang Q, Shi X, Wang Q, Zhang L, Wang X. 2020. Structure of the SARS-CoV-2 spike receptor-binding domain bound to the ACE2 receptor. Nature 581:215–220. 32225176 10.1038/s41586-020-2180-5

[7] Muecksch F, Weisblum Y, Barnes CO, Schmidt F, Schaefer-Babajew D, Wang Z, JC CL, Flyak AI, DeLaitsch AT, Huey-Tubman KE, Hou S, Schiffer CA, Gaebler C, Da Silva J, Poston D, Finkin S, Cho A, Cipolla M, Oliveira TY, Millard KG, Ramos V, Gazumyan A, Rutkowska M, Caskey M, Nussenzweig MC, Bjorkman PJ, Hatziioannou T, Bieniasz PD. 2021. Affinity maturation of SARS-CoV-2 neutralizing antibodies confers potency, breadth, and resilience to viral escape mutations. Immunity 54:1853–1868 e7. 34331873 10.1016/j.immuni.2021.07.008 PMC8323339

[8] Behrens AJ, Crispin M. 2017. Structural principles controlling HIV envelope glycosylation. Curr Opin Struct Biol 44:125–133. 28363124 10.1016/j.sbi.2017.03.008 PMC5513759

[9] Sanda M, Morrison L, Goldman R. 2021. N- and O-Glycosylation of the SARS-CoV-2 Spike Protein. Anal Chem 93:2003–2009. 33406838 10.1021/acs.analchem.0c03173 PMC7805595

[10] Casalino L, Gaieb Z, Goldsmith JA, Hjorth CK, Dommer AC, Harbison AM, Fogarty CA, Barros EP, Taylor BC, McLellan JS, Fadda E, Amaro RE. 2020. Beyond Shielding: The Roles of Glycans in the SARS-CoV-2 Spike Protein. ACS Cent Sci 6:1722–1734. 33140034 10.1021/acscentsci.0c01056 PMC7523240

[11] Zhao X, Chen H, Wang H. 2021. Glycans of SARS-CoV-2 Spike Protein in Virus Infection and Antibody Production. Front Mol Biosci 8:629873. 33928117 10.3389/fmolb.2021.629873 PMC8076860

[12] Grant OC, Montgomery D, Ito K, Woods RJ. 2020. Analysis of the SARS-CoV-2 spike protein glycan shield reveals implications for immune recognition. Sci Rep 10:14991. 32929138 10.1038/s41598-020-71748-7 PMC7490396

[13] Watanabe Y, Allen JD, Wrapp D, McLellan JS, Crispin M. 2020. Site-specific glycan analysis of the SARS-CoV-2 spike. Science 369:330–333. 32366695 10.1126/science.abb9983 PMC7199903

[14] Bagdonaite I, Thompson AJ, Wang X, Sogaard M, Fougeroux C, Frank M, Diedrich JK, Yates JR 3rd, Salanti A, Vakhrushev SY, Paulson JC, Wandall HH. 2021. Site-Specific O-Glycosylation Analysis of SARS-CoV-2 Spike Protein Produced in Insect and Human Cells. Viruses 13. 10.3390/v13040551 PMC8064498 33806155

[15] Shajahan A, Supekar NT, Gleinich AS, Azadi P. 2020. Deducing the N- and O-glycosylation profile of the spike protein of novel coronavirus SARS-CoV-2. Glycobiology 30:981–988. 32363391 10.1093/glycob/cwaa042 PMC7239183

[16] Tian W, Li D, Zhang N, Bai G, Yuan K, Xiao H, Gao F, Chen Y, Wong CCL, Gao GF. 2021. O-glycosylation pattern of the SARS-CoV-2 spike protein reveals an “O-Follow-N” rule. Cell Res 31:1123–1125. 34341488 10.1038/s41422-021-00545-2 PMC8326647

[17] Li Y, Liu D, Wang Y, Su W, Liu G, Dong W. 2021. The Importance of Glycans of Viral and Host Proteins in Enveloped Virus Infection. Front Immunol 12:638573. 33995356 10.3389/fimmu.2021.638573 PMC8116741

[18] Pollakis G, Kang S, Kliphuis A, Chalaby MI, Goudsmit J, Paxton WA. 2001. N-linked glycosylation of the HIV type-1 gp120 envelope glycoprotein as a major determinant of CCR5 and CXCR4 coreceptor utilization. J Biol Chem 276:13433–41. 11278567 10.1074/jbc.M009779200

[19] Wei X, Decker JM, Wang S, Hui H, Kappes JC, Wu X, Salazar-Gonzalez JF, Salazar MG, Kilby JM, Saag MS, Komarova NL, Nowak MA, Hahn BH, Kwong PD, Shaw GM. 2003. Antibody neutralization and escape by HIV-1. Nature 422:307–12. 12646921 10.1038/nature01470

[20] Reis CA, Tauber R, Blanchard V. 2021. Glycosylation is a key in SARS-CoV-2 infection. J Mol Med (Berl) 99:1023–1031. 34023935 10.1007/s00109-021-02092-0 PMC8140746

[21] Yang Q, Hughes TA, Kelkar A, Yu X, Cheng K, Park S, Huang WC, Lovell JF, Neelamegham S. 2020. Inhibition of SARS-CoV-2 viral entry upon blocking N- and O-glycan elaboration. Elife 9. 10.7554/eLife.61552 PMC7685702 33103998

[22] Sztain T, Ahn SH, Bogetti AT, Casalino L, Goldsmith JA, Seitz E, McCool RS, Kearns FL, Acosta-Reyes F, Maji S, Mashayekhi G, McCammon JA, Ourmazd A, Frank J, McLellan JS, Chong LT, Amaro RE. 2021. A glycan gate controls opening of the SARS-CoV-2 spike protein. Nat Chem 13:963–968. 34413500 10.1038/s41557-021-00758-3 PMC8488004

[23] Li Q, Wu J, Nie J, Zhang L, Hao H, Liu S, Zhao C, Zhang Q, Liu H, Nie L, Qin H, Wang M, Lu Q, Li X, Sun Q, Liu J, Zhang L, Li X, Huang W, Wang Y. 2020. The Impact of Mutations in SARS-CoV-2 Spike on Viral Infectivity and Antigenicity. Cell 182:1284–1294 e9. 32730807 10.1016/j.cell.2020.07.012 PMC7366990

[24] Barnes CO, Jette CA, Abernathy ME, Dam KA, Esswein SR, Gristick HB, Malyutin AG, Sharaf NG, Huey-Tubman KE, Lee YE, Robbiani DF, Nussenzweig MC, West AP Jr., Bjorkman PJ. 2020. SARS-CoV-2 neutralizing antibody structures inform therapeutic strategies. Nature 588:682–687. 33045718 10.1038/s41586-020-2852-1 PMC8092461

[25] Pinto D, Park YJ, Beltramello M, Walls AC, Tortorici MA, Bianchi S, Jaconi S, Culap K, Zatta F, De Marco A, Peter A, Guarino B, Spreafico R, Cameroni E, Case JB, Chen RE, Havenar-Daughton C, Snell G, Telenti A, Virgin HW, Lanzavecchia A, Diamond MS, Fink K, Veesler D, Corti D. 2020. Cross-neutralization of SARS-CoV-2 by a human monoclonal SARS-CoV antibody. Nature 583:290–295. 32422645 10.1038/s41586-020-2349-y

[26] Fang Y, Sun P, Xie X, Du M, Du F, Ye J, Kalveram BK, Plante JA, Plante KS, Li B, Bai XC, Shi PY, Chen ZJ. 2022. An antibody that neutralizes SARS-CoV-1 and SARS-CoV-2 by binding to a conserved spike epitope outside the receptor binding motif. Sci Immunol 7:eabp9962. 35926067 10.1126/sciimmunol.abp9962 PMC9407945

[27] Crawford KHD, Eguia R, Dingens AS, Loes AN, Malone KD, Wolf CR, Chu HY, Tortorici MA, Veesler D, Murphy M, Pettie D, King NP, Balazs AB, Bloom JD. 2020. Protocol and Reagents for Pseudotyping Lentiviral Particles with SARS-CoV-2 Spike Protein for Neutralization Assays. Viruses 12. 10.3390/v12050513 PMC7291041 32384820

[28] Schmidt F, Weisblum Y, Muecksch F, Hoffmann HH, Michailidis E, Lorenzi JCC, Mendoza P, Rutkowska M, Bednarski E, Gaebler C, Agudelo M, Cho A, Wang Z, Gazumyan A, Cipolla M, Caskey M, Robbiani DF, Nussenzweig MC, Rice CM, Hatziioannou T, Bieniasz PD. 2020. Measuring SARS-CoV-2 neutralizing antibody activity using pseudotyped and chimeric viruses. J Exp Med 217. 10.1084/jem.20201181 PMC7372514 32692348

[29] Weisblum Y, Schmidt F, Zhang F, DaSilva J, Poston D, Lorenzi JC, Muecksch F, Rutkowska M, Hoffmann HH, Michailidis E, Gaebler C, Agudelo M, Cho A, Wang Z, Gazumyan A, Cipolla M, Luchsinger L, Hillyer CD, Caskey M, Robbiani DF, Rice CM, Nussenzweig MC, Hatziioannou T, Bieniasz PD. 2020. Escape from neutralizing antibodies by SARS-CoV-2 spike protein variants. Elife 9. 10.7554/eLife.61312 PMC7723407 33112236

[30] Christensen DE, Ganser-Pornillos BK, Johnson JS, Pornillos O, Sundquist WI. 2020. Reconstitution and visualization of HIV-1 capsid-dependent replication and integration in vitro. Science 370. 10.1126/science.abc8420 PMC8022914 33033190

[31] Briggs JA, Simon MN, Gross I, Kräusslich HG, Fuller SD, Vogt VM, Johnson MC. 2004. The stoichiometry of Gag protein in HIV-1. Nat Struct Mol Biol 11:672–5. 15208690 10.1038/nsmb785

[32] Klein S, Cortese M, Winter SL, Wachsmuth-Melm M, Neufeldt CJ, Cerikan B, Stanifer ML, Boulant S, Bartenschlager R, Chlanda P. 2020. SARS-CoV-2 structure and replication characterized by in situ cryo-electron tomography. Nat Commun 11:5885. 33208793 10.1038/s41467-020-19619-7 PMC7676268

[33] Zhu P, Chertova E, Bess J Jr., Lifson JD, Arthur LO, Liu J, Taylor KA, Roux KH. 2003. Electron tomography analysis of envelope glycoprotein trimers on HIV and simian immunodeficiency virus virions. Proc Natl Acad Sci U S A 100:15812–7. 14668432 10.1073/pnas.2634931100 PMC307650

[34] Robbiani DF, Gaebler C, Muecksch F, Lorenzi JCC, Wang Z, Cho A, Agudelo M, Barnes CO, Gazumyan A, Finkin S, Hagglof T, Oliveira TY, Viant C, Hurley A, Hoffmann HH, Millard KG, Kost RG, Cipolla M, Gordon K, Bianchini F, Chen ST, Ramos V, Patel R, Dizon J, Shimeliovich I, Mendoza P, Hartweger H, Nogueira L, Pack M, Horowitz J, Schmidt F, Weisblum Y, Michailidis E, Ashbrook AW, Waltari E, Pak JE, Huey-Tubman KE, Koranda N, Hoffman PR, West AP Jr., Rice CM, Hatziioannou T, Bjorkman PJ, Bieniasz PD, Caskey M, Nussenzweig MC. 2020. Convergent antibody responses to SARS-CoV-2 in convalescent individuals. Nature 584:437–442. 32555388 10.1038/s41586-020-2456-9 PMC7442695

[35] Gaebler C, Wang Z, Lorenzi JCC, Muecksch F, Finkin S, Tokuyama M, Cho A, Jankovic M, Schaefer-Babajew D, Oliveira TY, Cipolla M, Viant C, Barnes CO, Bram Y, Breton G, Hagglof T, Mendoza P, Hurley A, Turroja M, Gordon K, Millard KG, Ramos V, Schmidt F, Weisblum Y, Jha D, Tankelevich M, Martinez-Delgado G, Yee J, Patel R, Dizon J, Unson-O’Brien C, Shimeliovich I, Robbiani DF, Zhao Z, Gazumyan A, Schwartz RE, Hatziioannou T, Bjorkman PJ, Mehandru S, Bieniasz PD, Caskey M, Nussenzweig MC. 2021. Evolution of antibody immunity to SARS-CoV-2. Nature 591:639–644. 33461210 10.1038/s41586-021-03207-w PMC8221082

[36] Wang Z, Muecksch F, Schaefer-Babajew D, Finkin S, Viant C, Gaebler C, Hoffmann HH, Barnes CO, Cipolla M, Ramos V, Oliveira TY, Cho A, Schmidt F, Da Silva J, Bednarski E, Aguado L, Yee J, Daga M, Turroja M, Millard KG, Jankovic M, Gazumyan A, Zhao Z, Rice CM, Bieniasz PD, Caskey M, Hatziioannou T, Nussenzweig MC. 2021. Naturally enhanced neutralizing breadth against SARS-CoV-2 one year after infection. Nature 595:426–431. 34126625 10.1038/s41586-021-03696-9 PMC8277577

[37] Jette CA, Cohen AA, Gnanapragasam PNP, Muecksch F, Lee YE, Huey-Tubman KE, Schmidt F, Hatziioannou T, Bieniasz PD, Nussenzweig MC, West AP Jr., Keeffe JR, Bjorkman PJ, Barnes CO. 2021. Broad cross-reactivity across sarbecoviruses exhibited by a subset of COVID-19 donor-derived neutralizing antibodies. Cell Rep 36:109760. 34534459 10.1016/j.celrep.2021.109760 PMC8423902

[38] Magnus C, Rusert P, Bonhoeffer S, Trkola A, Regoes RR. 2009. Estimating the stoichiometry of human immunodeficiency virus entry. J Virol 83:1523–31. 19019953 10.1128/JVI.01764-08 PMC2620894

[39] Watanabe Y, Bowden TA, Wilson IA, Crispin M. 2019. Exploitation of glycosylation in enveloped virus pathobiology. Biochim Biophys Acta Gen Subj 1863:1480–1497. 31121217 10.1016/j.bbagen.2019.05.012 PMC6686077

[40] Allen JD, Ivory DP, Song SG, He WT, Capozzola T, Yong P, Burton DR, Andrabi R, Crispin M. 2023. The diversity of the glycan shield of sarbecoviruses related to SARS-CoV-2. Cell Rep 42:112307. 36972173 10.1016/j.celrep.2023.112307 PMC10015101

[41] Shajahan A, Pepi L, Kumar B, Murray N, Azadi P. 2023. Site Specific N- and O-glycosylation mapping of the Spike Proteins of SARS-CoV-2 Variants of Concern. Sci Rep 13:10053. 37344512 10.1038/s41598-023-33088-0 PMC10284906

[42] Mori T, Jung J, Kobayashi C, Dokainish HM, Re S, Sugita Y. 2021. Elucidation of interactions regulating conformational stability and dynamics of SARS-CoV-2 S-protein. Biophys J 120:1060–1071. 33484712 10.1016/j.bpj.2021.01.012 PMC7825899

[43] Dokainish HM, Re S, Mori T, Kobayashi C, Jung J, Sugita Y. 2022. The inherent flexibility of receptor binding domains in SARS-CoV-2 spike protein. Elife 11. 10.7554/eLife.75720 PMC8963885 35323112

[44] Pang YT, Acharya A, Lynch DL, Pavlova A, Gumbart JC. 2022. SARS-CoV-2 spike opening dynamics and energetics reveal the individual roles of glycans and their collective impact. Commun Biol 5:1170. 36329138 10.1038/s42003-022-04138-6 PMC9631587

[45] Cho A, Muecksch F, Schaefer-Babajew D, Wang Z, Finkin S, Gaebler C, Ramos V, Cipolla M, Mendoza P, Agudelo M, Bednarski E, DaSilva J, Shimeliovich I, Dizon J, Daga M, Millard KG, Turroja M, Schmidt F, Zhang F, Tanfous TB, Jankovic M, Oliveria TY, Gazumyan A, Caskey M, Bieniasz PD, Hatziioannou T, Nussenzweig MC. 2021. Anti-SARS-CoV-2 receptor-binding domain antibody evolution after mRNA vaccination. Nature 600:517–522. 34619745 10.1038/s41586-021-04060-7 PMC8674133

