# Supplementary material for: SARS-CoV-2 spike glycosylation affects function and neutralization sensitivity
Source: bioRxiv. 2023 Jun 30:2023.06.30.547241. Preprint. [Version 1] doi: 10.1101/2023.06.30.547241 (PMC10327196; doi:10.1101/2023.06.30.547241)
Supplement: Supplement 1 [file NIHPP2023.06.30.547241v1-supplement-1.pdf]

bioRxiv preprint doi: https://doi.org/10.1101/2023.06.30.547241; this version posted June 30, 2023. The copyright holder for this preprint (which was not certified by peer review) is the author/funder, who has granted bioRxiv a license to display the preprint in perpetuity. It is made available under aCC-BY 4.0 International license.

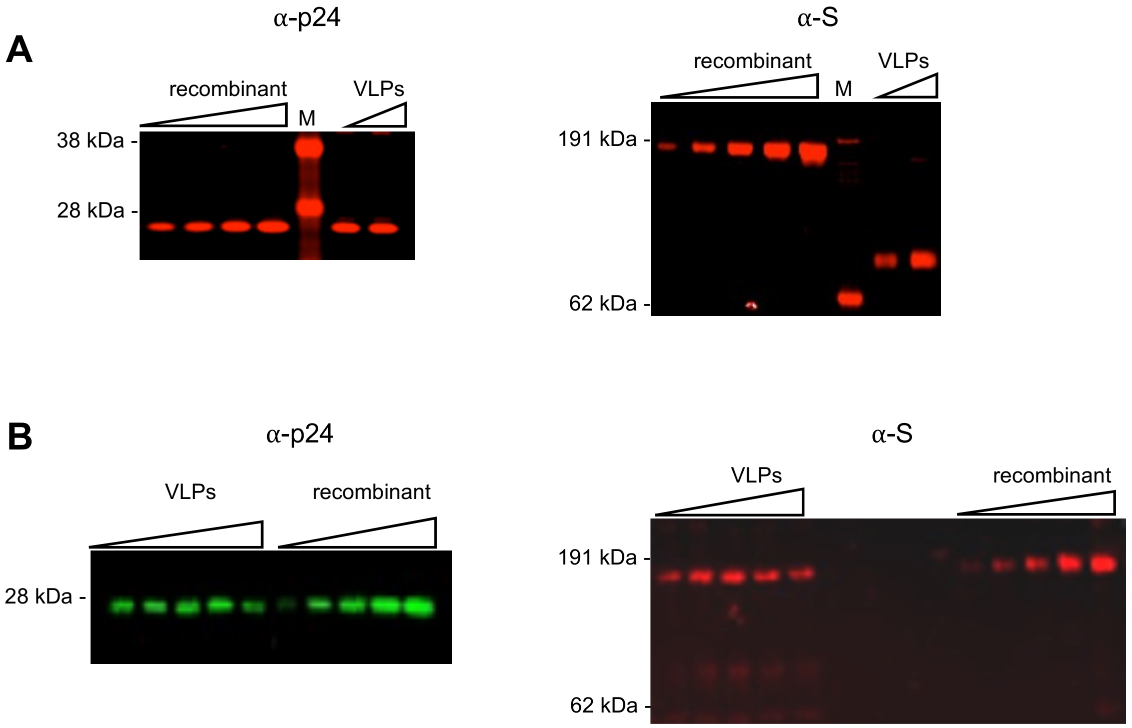

FIG S1 Western blot analysis of virions, using recombinant proteins as standard
(A) Western blot analysis of virions pelleted through 20% sucrose from 100 µl supernatant harvested at 48 hours after transfection with 0.0625 µg or 0.5 µg of wild-type spike expression plasmid along with envelope-deficient HIV-1 proviral plasmid expressing NanoLuc luciferase. The blot was probed with an anti-p24 antibody and recombinant HIV p24 protein was used as a standard (1.0 ng, 2.0 ng, 4.0 ng, or 8.0 ng per lane) on the left, or with anti-Spike antibody using recombinant S-6P-nanoLuc as a standard (0.25 ng, 0.5 ng, 1.0 ng, 2.0 ng, or 4.0 ng per lane) on the right. Representative of two independent experiments.
(B) Western blot analysis of virions pelleted through 20% sucrose from 100 µl supernatant harvested at 48 hours after transfection with 0.008 µg, 0.024 µg, 0.073 µg, 0.22 µg, or 0.67 µg of wild-type spike expression (furin uncleavable R683G background) along with envelope-deficient HIV-1 proviral plasmid expressing NanoLuc luciferase. The blot was probed with anti-p24 antibody using recombinant HIV p24 protein as standard (0.5 ng, 1.0 ng, 2.0 ng, 4.0 ng, or 8.0 ng per lane) on the left, or with anti-Spike antibody using recombinant S-6P-nanoLuc as standard (0.125 ng, 0.25 ng, 0.5 ng, 1.0 ng, or 2.0 ng per lane) on the right. Representative of two independent experiments.

bioRxiv preprint doi: https://doi.org/10.1101/2023.06.30.547241; this version posted June 30, 2023. The copyright holder for this preprint (which was not certified by peer review) is the author/funder, who has granted bioRxiv a license to display the preprint in perpetuity. It is made available under aCC-BY 4.0 International license.

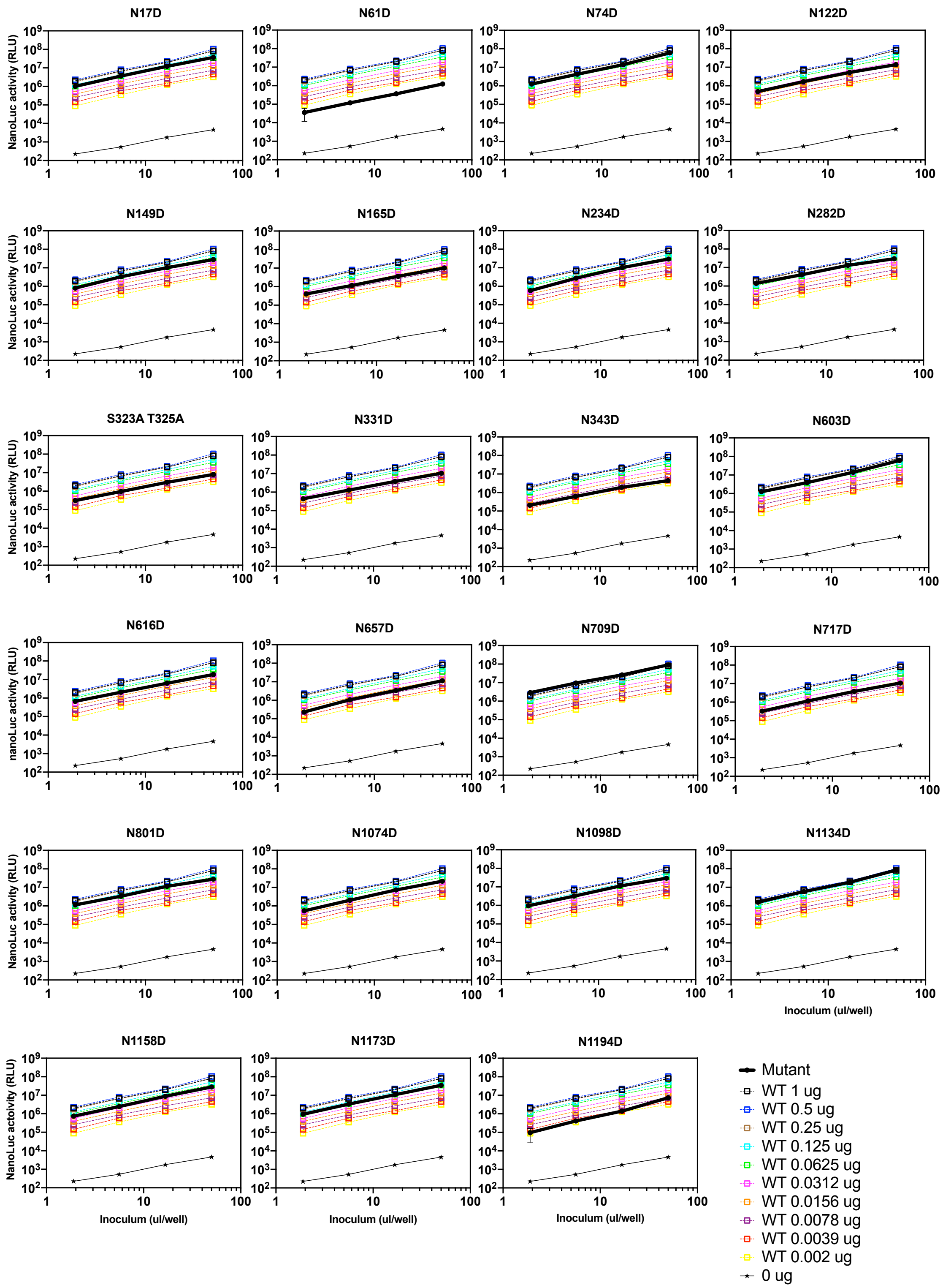

FIG S2 The impact of glycosylation site mutations on particle infectivity

Infectious virion measurements of for pseudotypes bearing glycosylation site mutant spike proteins (bold black lines, 1 µg transfected S expression plasmid), compared with as well as WT S pseudoytpes (dashed lines) collected from 293T cells transfected with various amounts of spike expression plasmids. Infection was quantified by measuring

bioRxiv preprint doi: https://doi.org/10.1101/2023.06.30.547241; this version posted June 30, 2023. The copyright holder for this preprint (which was not certified by peer review) is the author/funder, who has granted bioRxiv a license to display the preprint in perpetuity. It is made available under aCC-BY 4.0 International license.

NanoLuc luciferase activity (RLU). Virus generated in the absence of S (0 μg), shown in thin black line, was used as a background control. 293T/ACE2.cl22, as target cells, were infected with the indicated volumes of pseudotyped viruses in 96-well plates and harvested 48 hours post infection for NanoLuc luciferase assay. The mean and range deviation from two technical replicates are shown.

bioRxiv preprint doi: https://doi.org/10.1101/2023.06.30.547241; this version posted June 30, 2023. The copyright holder for this preprint (which was not certified by peer review) is the author/funder, who has granted bioRxiv a license to display the preprint in perpetuity. It is made available under aCC-BY 4.0 International license.

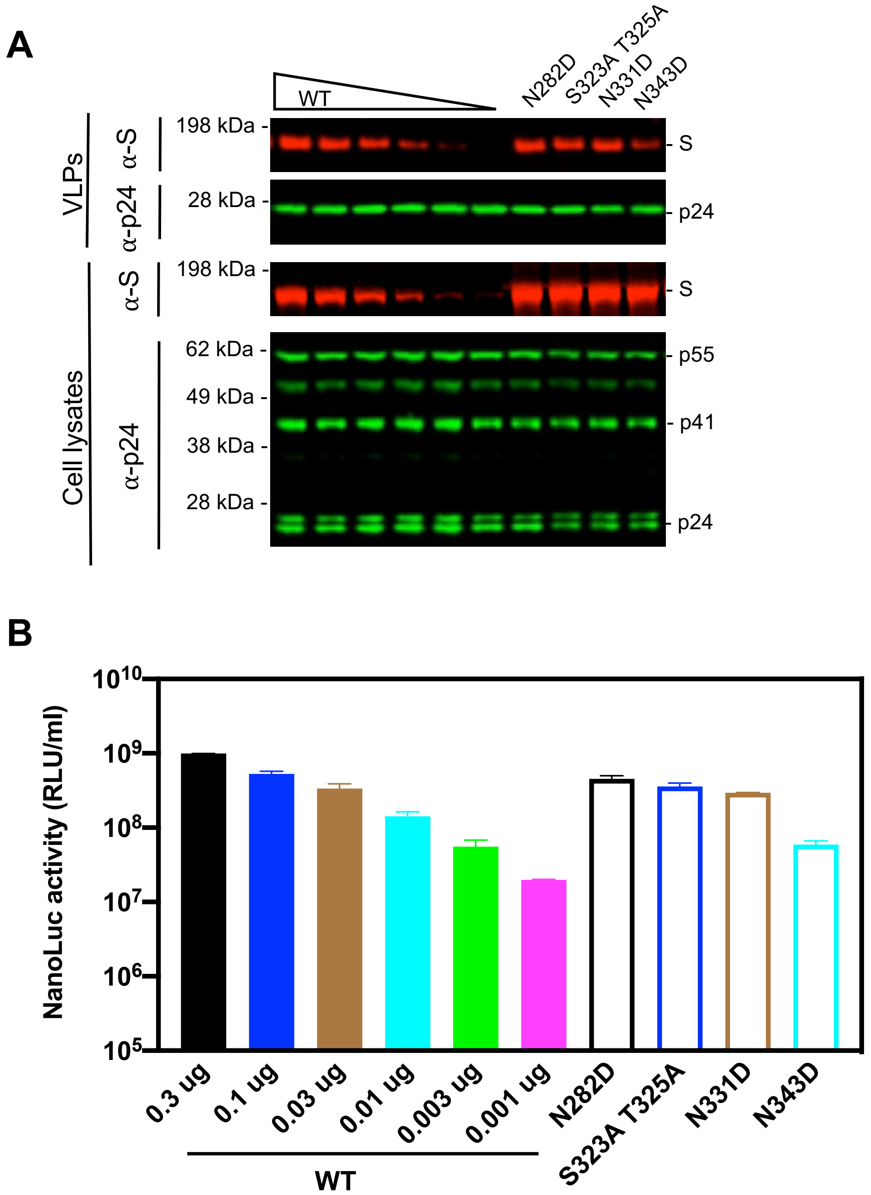

FIG S3 The impact of glycosylation site mutations in the furin uncleavable (R683G) background on spike incorporation and particle infectivity
(A) Western blot analysis of 293T cell lysates (lower panel) or virions (upper panel) at 48 hours after transfection with various amounts of glycosylation site intact spike expression plasmid (R683G background), or 1 μg of glycosylation site mutants (N282D, S323A T325A, N331D, or N343D) along with envelope-deficient HIV-1 proviral plasmid expressing NanoLuc.
(B) Infectivity was quantified by measuring NanoLuc luciferase activity (RLUs) following infection of 293T expressing ACE2 (293T/ACE2.cl22) in 96-well plates with pseudotyped viruses as depicted in (A). The mean and range of two technical replicates are plotted.

bioRxiv preprint doi: https://doi.org/10.1101/2023.06.30.547241; this version posted June 30, 2023. The copyright holder for this preprint (which was not certified by peer review) is the author/funder, who has granted bioRxiv a license to display the preprint in perpetuity. It is made available under aCC-BY 4.0 International license.

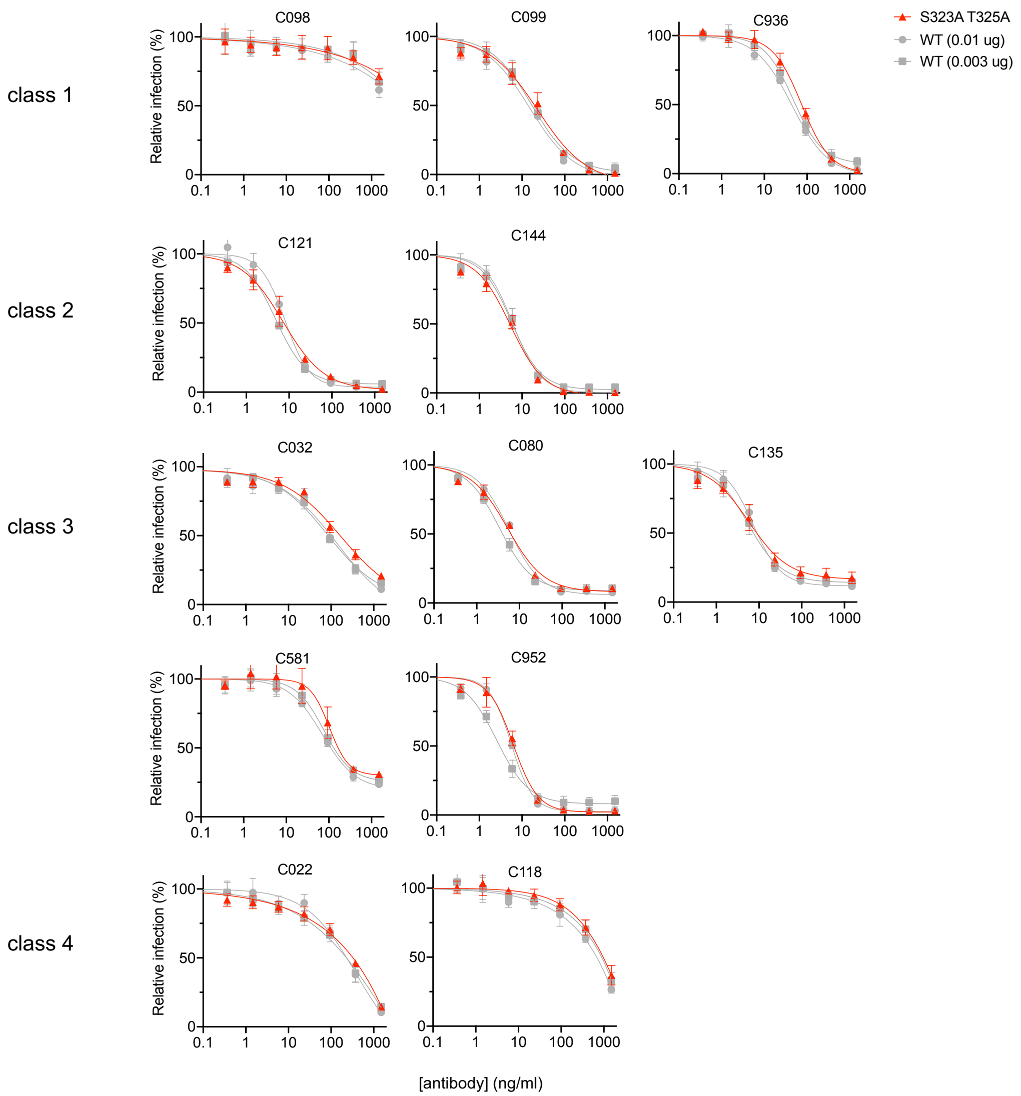

FIG S4 Mutations of the O-linked glycosylation sites at 323 and 325 in the RBD (S323A T325A) have marginal effect on neutralization sensitivity
Neutralization of glycosylation site mutant S323A T325A pseudotyped virus infection in the presence of the indicated concentrations of a panel of monoclonal antibodies, including class 1 (C098, C099, and C936), class 2 (C121 and C144), class 3 (C032, C080, C135, C581, and C952), and class 4 (C022 and C118) antibodies. As controls, glycosylation intact spike expression plasmid (WT in the furin uncleavable R683G background) was transfected at two doses, 10 ng or 3 ng, and the resulting viruses were assessed for neutralization sensitivity in parallel. The mean and range of two technical replicates are shown.

bioRxiv preprint doi: https://doi.org/10.1101/2023.06.30.547241; this version posted June 30, 2023. The copyright holder for this preprint (which was not certified by peer review) is the author/funder, who has granted bioRxiv a license to display the preprint in perpetuity. It is made available under aCC-BY 4.0 International license.

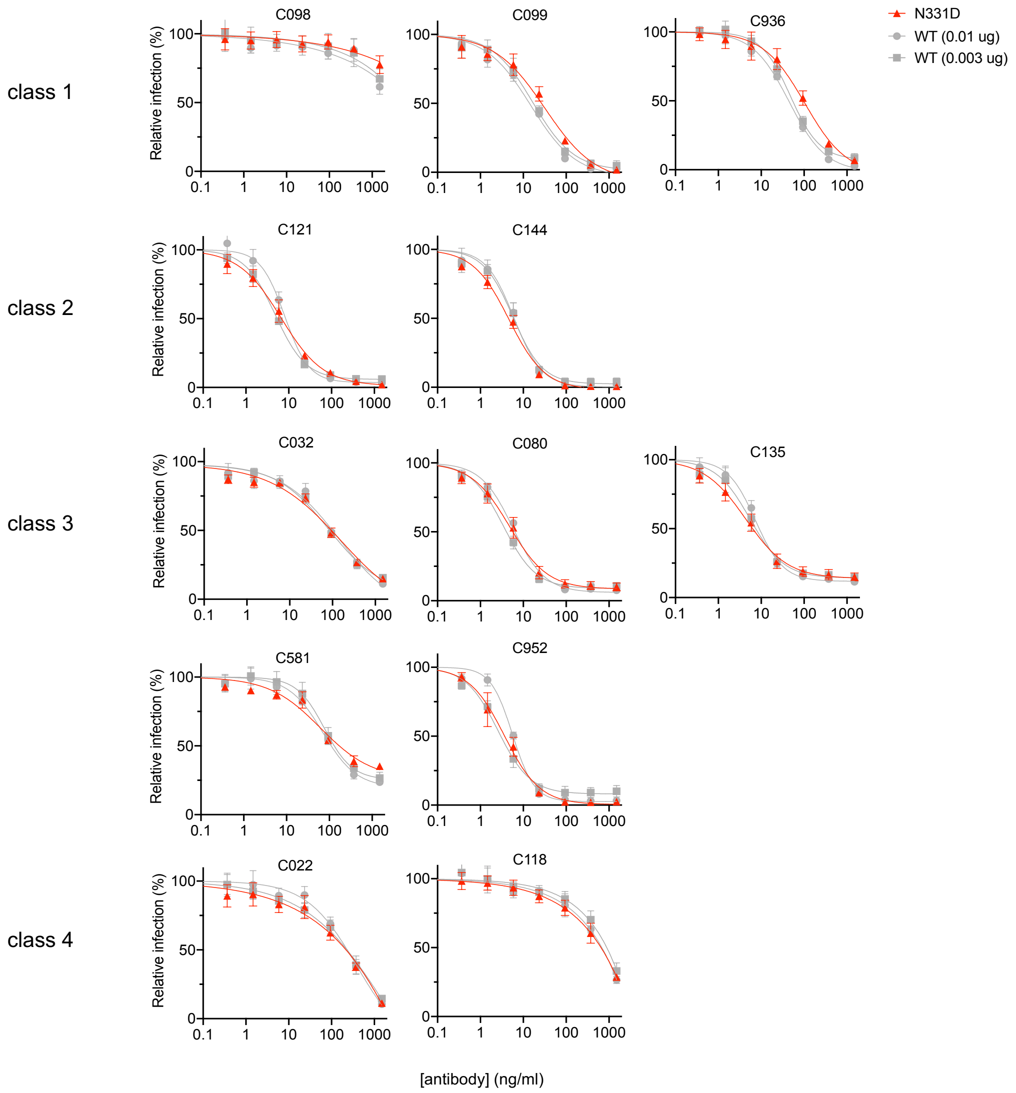

FIG S5 Effect of glycosylation at N331 on neutralization sensitivity
Neutralization of glycosylation site mutant N331D pseudotyped virus infection in the presence of the indicated concentrations of a panel of monoclonal antibodies, including class 1 (C098, C099, and C936), class 2 (C121 and C144), class 3 (C032, C080, C135, C581, and C952), and class 4 (C022 and C118) antibodies. As controls, glycosylation intact spike (WT in the furin uncleavable R683G background) was transfected at two doses, 10 ng or 3 ng, and the resulting viruses were assessed for neutralization sensitivity in parallel. The mean and range of two technical replicates are shown.

bioRxiv preprint doi: https://doi.org/10.1101/2023.06.30.547241; this version posted June 30, 2023. The copyright holder for this preprint (which was not certified by peer review) is the author/funder, who has granted bioRxiv a license to display the preprint in perpetuity. It is made available under aCC-BY 4.0 International license.

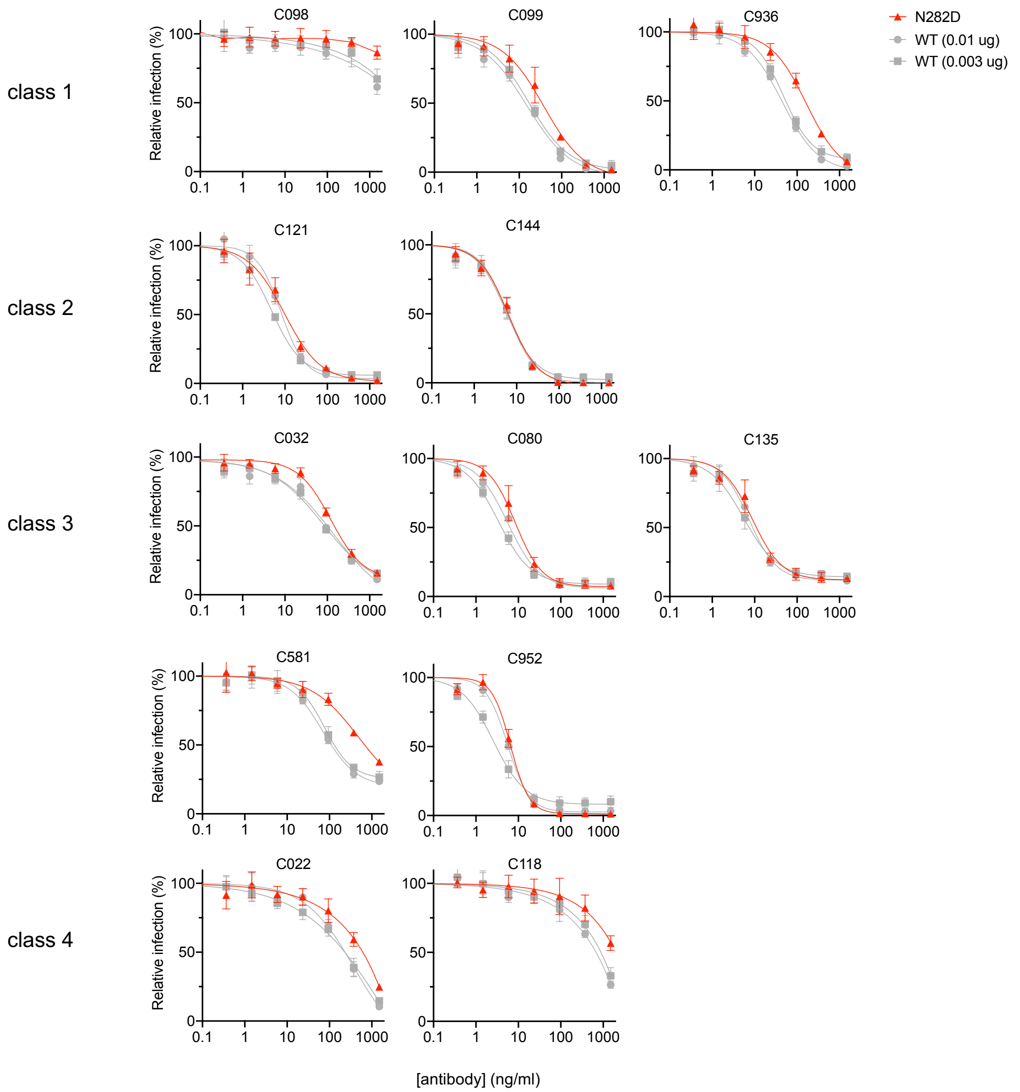

FIG S6 Effect of glycosylation at N282 on neutralization sensitivity
Neutralization of glycosylation site mutant N282D pseudotyped virus infection in the presence of the indicated concentrations of a panel of monoclonal antibodies, including class 1 (C098, C099, and C936), class 2 (C121 and C144), class 3 (C032, C080, C135, C581, and C952), and class 4 (C022 and C118). As controls, glycosylation intact spike (WT in the furin uncleavable R683G background) was transfected at two doses, 10 ng or 3 ng, and the resulting viruses were assessed for neutralization sensitivity in parallel. The mean and range of two technical replicates are shown.

bioRxiv preprint doi: https://doi.org/10.1101/2023.06.30.547241; this version posted June 30, 2023. The copyright holder for this preprint (which was not certified by peer review) is the author/funder, who has granted bioRxiv a license to display the preprint in perpetuity. It is made available under aCC-BY 4.0 International license.

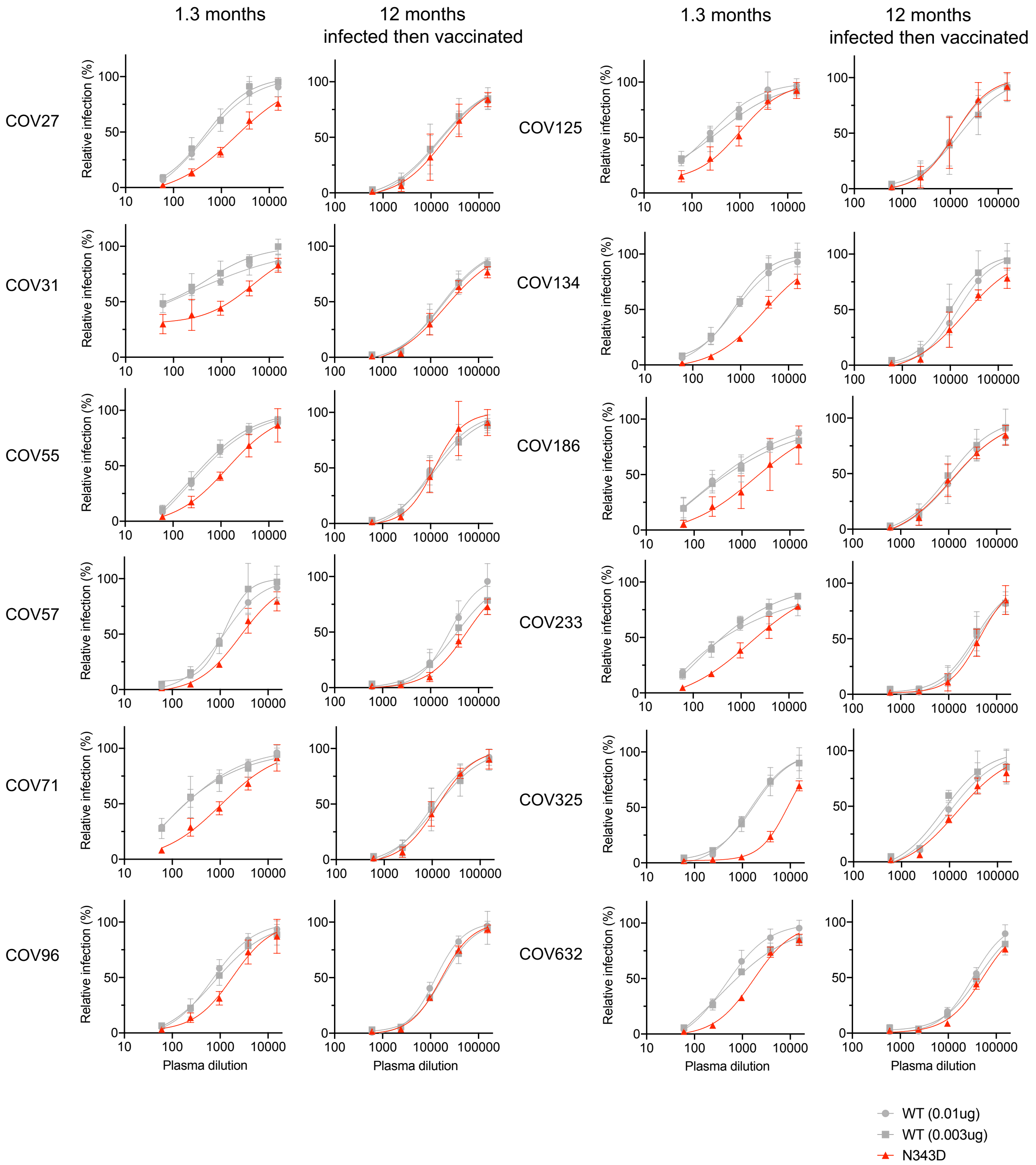

FIG S7 Neutralization sensitivity of N343D mutant to convalescent plasma
Additional examples of plasma neutralization of N343D or glycosylation site intact spike (in the furin uncleavable R683G background, same as FIG 5) pseudotyped virus using 293T/ACE2.cl22 target cells. The mean and range of two technical replicates are shown.
